# Type 1 Interferons Inhibit Myotube Formation Independently of Upregulation of Interferon-Stimulated Gene 15

**DOI:** 10.1371/journal.pone.0065362

**Published:** 2013-06-04

**Authors:** Sara Franzi, Mohammad Salajegheh, Remedios Nazareno, Steven A. Greenberg

**Affiliations:** 1 Children’s Hospital Informatics Program, Boston Children’s Hospital, Boston, Massachusetts, United States of America; 2 Department of Neurology IV, Neuromuscular Diseases and Neuroimmunology, Fondazione Istituto Neurologico ‘Carlo Besta’, Milan, Italy; 3 Department of Neurology, Brigham and Women’s Hospital, Boston, Massachusetts, United States of America; 4 Harvard Medical School, Boston, Massachusetts, United States of America; University of Jaén, Spain

## Abstract

**Introduction:**

Type 1 interferon (IFN)-inducible genes and their inducible products are upregulated in dermatomyositis muscle. Of these, IFN-stimulated gene 15 (ISG15) is one of the most upregulated, suggesting its possible involvement in the pathogenesis of this disease. To test this postulate, we developed a model of type 1 IFN mediated myotube toxicity and assessed whether or not downregulation of ISG15 expression prevents this toxicity.

**Methods:**

Mouse myoblasts (C2C12 cell line) were cultured in the presence of type 1 or type 2 IFNs and ISG15 expression assessed by microarray analysis. The morphology of newly formed myotubes was assessed by measuring their length, diameter, and area on micrographs using imaging software. ISG15 expression was silenced through transfection with small interference RNA.

**Results:**

Type 1 IFNs, especially IFN-beta, increased ISG15 expression in C2C12 cells and impaired myotube formation. Silencing of ISG15 resulted in knockdown of ISG15 protein, but without phenotypic rescue of myotube formation.

**Discussion:**

IFN-beta affects myoblast differentiation ability and myotube morphology in vitro.These studies provide evidence that ISG15, which is highly upregulated in dermatomyositis muscle, does not appear to play a key role in IFN-beta-mediated C2C12 myoblast cell fusion.

## Introduction

Binding of type 1 interferons (IFNs), which include IFN-α and IFN-β, to type 1 interferon receptor on target cells stimulates the transcription and translation of a set of genes known as the type 1 IFN-inducible genes. Proteins produced from these genes’ transcripts, such as IFN-stimulated gene 15 (ISG15) and myxovirus resistance protein A (MxA), play a role in defending cells from viral and bacterial infections and are part of the innate immune system.

Type 1 IFN-inducible genes, including ISG15, are highly upregulated in muscle [1]–[6], blood [4], [7], and skin [8] of patients with dermatomyositis (DM), an autoimmune disease affecting skeletal muscle and other tissues. Endothelial tubuloreticular inclusions and the proteins MxA and ISG15 are found in abundance intracellularly in diseased myofibers, keratinocytes, and capillaries of DM muscle and skin [3], [5], [9]. Plasmacytoid dendritic cells (pDCs), professional type 1 interferon producing cells, are abundant in DM muscle and skin [3], [10], [11]. IFN-β protein in serum [12] and IFN-β transcript in skin [7] are elevated in DM and correlate with a type 1 interferon gene expression signature. In endothelial cell culture models, tubuloreticular inclusions are induced by type 1, but not type 2 (consisting of the sole member IFN-γ), IFN exposure [13]–[16]. In human skeletal muscle cells (HuSK), ISG15 gene and protein expression are highly induced by IFN-β [5]. Together, these findings suggest that exposure of relevant cells in culture to type 1 IFN could be a suitable model to study possible mechanisms of myofiber and capillary injury in DM driven by type 1 IFNs.

In this study therefore, we have used the C2C12 mouse myoblast cell line to examine the possible effect of type 1 IFNs on myotube formation. Because ISG15 is one of the most upregulated genes in DM and ISG15 protein localizes by immunohistochemistry to atrophic myofibers [5], we examined its possible role in IFN-mediated myotoxicity in vitro.

## Results

### Type 1 IFNs Upregulate ISG15 in C2C12 Mouse Myoblasts

**Figure 1:**
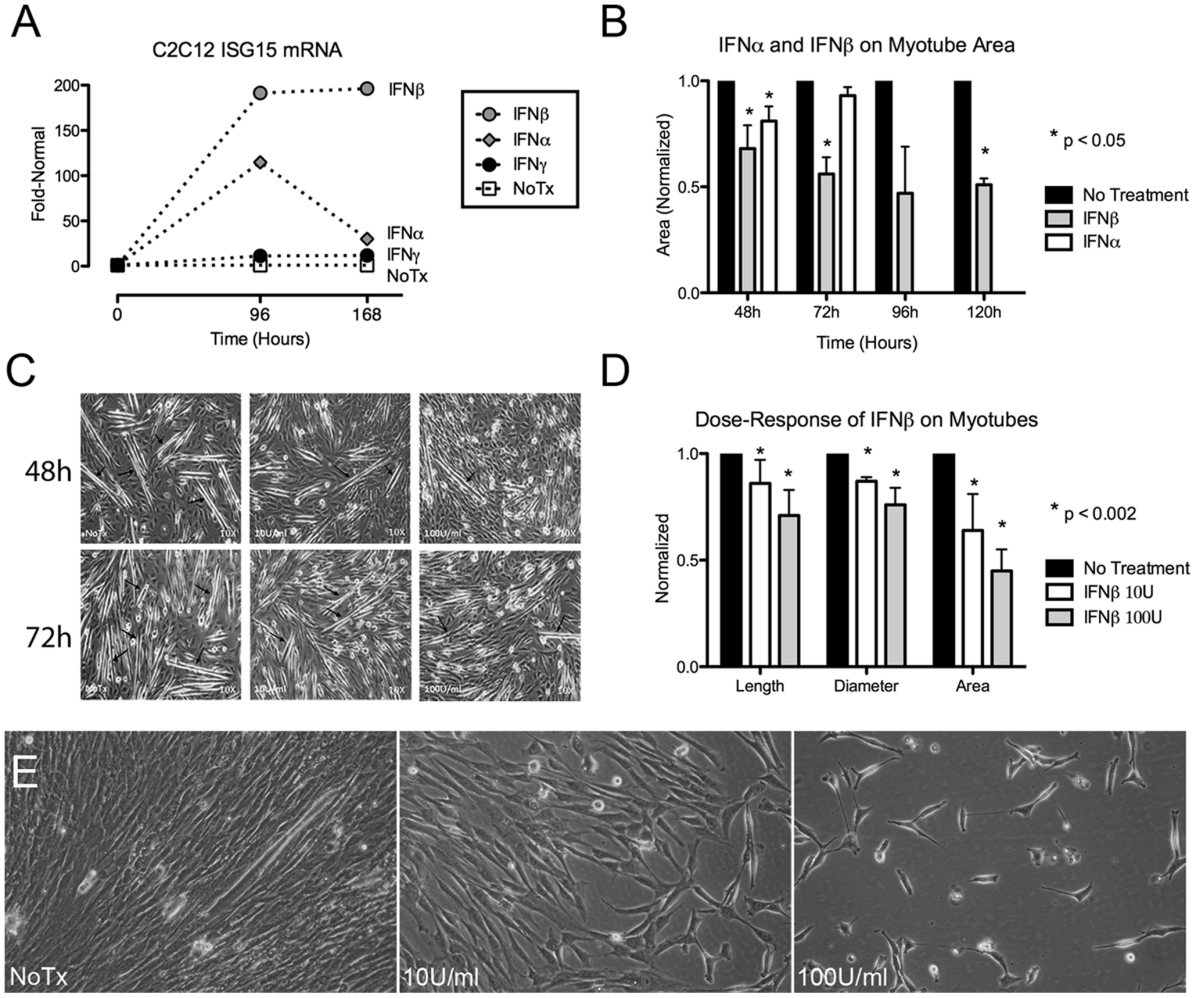
Effects of type 1 IFNs on mouse C2C12 and human muscle cells. (A) IFN-β results in sustained marked expression of ISG15 (196-fold increased at Day 7). (B) Sustained toxicity of IFN-β on myotube area. (C–E) Dose-dependent effects of IFN-β 10 U/ml and 100 U/ml on myotubes. (C) Dose-dependent reduction in numbers and lengths of C2C12 myotubes at 48 h and 72 h. Arrows indicate myotubes. (D) Dose-dependent reduction in C2C12 myotube length, diameter, and area at 72h. (E) Dose-dependent effect of IFN-β on 72 h human skeletal muscle with marked inhibition of myotube formation at 100 U/ml.

In previously published studies, ISG15 was upregulated 194-fold in human DM muscle biopsy samples [5]. We studied a muscle cell culture line, C2C12 cells, stimulating them with IFN-α, IFN-β, and IFN-γ for 7 days and assessed global transcriptional responses at Day 4 and Day 7 (manuscript in preparation). ISG15 gene expression was upregulated on Day 4 114-fold in response to IFN-α, 191-fold in response to IFN-β, and 11-fold in response to IFN-γ (Figure 1A). ISG15’s marked upregulation by IFN-β was sustained at Day 7 (196-fold) in contrast to its response to IFN-α that had diminished compared to Day 4 (30-fold).

### Type 1 IFNs Impair the Differentiation of C2C12 Mouse Myoblasts and Human Skeletal Muscle

These data prompted us to further investigate the role of type 1 IFNs during myoblast differentiation. We initially focused on early time points (48 h and 72 h) because of the greater uniformity of early myoblast differentiation. Treatment of cultured C2C12 mouse myoblasts with type 1 IFNs resulted in significant alteration in the timing of differentiation and in the morphology of new myotubes, as compared to untreated cells. Untreated cells started to differentiate before 48 h in low-serum medium, while type 1 interferon treatment impaired myoblast differentiation into myotubes. Myotube areas (reflecting both mean diameter and length) at 48 hours were decreased 32% by IFN-β (p<0.0001) and 19% by IFN-α (p<0.0001) compared to untreated myotubes (Figure 1B). At 72 hours, the inhibitory effect of IFN-β remained (44% reduction in area; p<0.0001), whereas the inhibitory effects of IFN-α were no longer present (7%; p = 0.74). These sustained effects of IFN-β on myotube development, together with transcriptional data indicating sustained effects of IFN-β on ISG15 upregulation and recent findings implicating IFN-β in the pathogenesis of dermatomyositis [8], [12], led us to focus further experiments on IFN-β alone. We therefore extended quantitative studies on IFN-β’s effect to 96 h and 120 h and observed sustained impairment in myotube morphology (e.g., 49% decreased area, p = 0.02, at 120 h; Figure 1B).

We next conducted dose-response studies of IFN-β’s effect on myotube length, diameter, and area (Figure 1C, D). At 48 h and 72 h, IFN-β at doses of 10 U/ml and 100 U/ml visibly decreased numbers of myotube formation in a dose-dependent manner, with the larger dose resulting in fewer and shorter myotubes (Figure 1C). Quantitative analysis showed similar dose-dependent relationships on myotube length, diameter, and area (e.g., myotube area decreased 36% with 10 U/ml and 55% with 100 U/ml, p<0.002; Figure 1D).

Lastly, we examined human skeletal muscle cell culture and found dose-dependent marked toxicity of IFN-β, with 100 U/ml completely preventing myotube formation at 48 h and 72 h (Figure 1E). Similarly to C2C12, IFN-α was considerably less toxic to HuSK (data not shown).

### Silencing ISG15 does not Prevent IFN-β-mediated Toxicity for C2C12 Myoblasts

**Figure 2:**
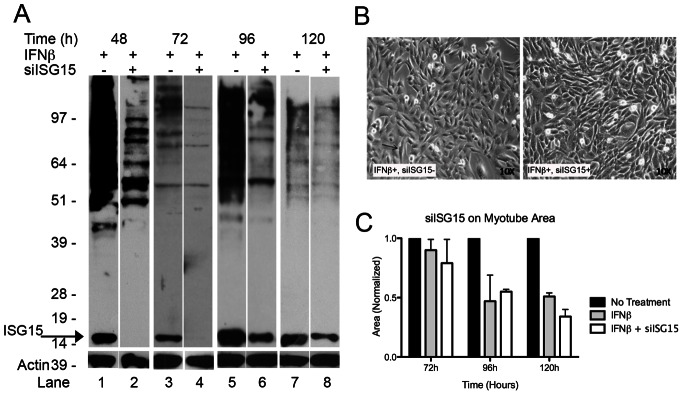
RNA silencing of ISG15 does not prevent IFN-β mediated myotoxicity. (A) Western blots of ISG15 demonstrate successful silencing of the 15 kDa ISG15 protein with siISG15 treatment in IFN-β treated C2C12 cells. Note absence of 15 kDa bands at 48 h (lane 2 compared to lane 1) and 72 h (lane 4 compared to lane 3), with partial return of protein expression at 96 h (lanes 6/5) and 120 h (lanes 8/7). siISG15 also reduces ISG15 conjugates (smears of >50 kDa) at all time points. Actin controls shown below. (B) Images demonstrate no improvement in myotube formation with ISG15 silencing at 72 h, and (C) quantitative analysis shows ISG15 silencing with siISG15 results in no recovery of myotube area at 72 h, 96 h, and 120 h.

To assess whether the toxic effect of IFN-β on the differentiation of C2C12 myoblasts was mediated by ISG15, we silenced ISG15 by transfecting C2C12 myoblasts with siRNA against ISG15. ISG15 protein expression, as assessed by Western blotting, is highly increased in type 1 IFN-treated HuSK muscle cells in vitro and in DM patient muscle [2]. We therefore assessed C2C12 ISG15 protein expression by Western blotting at different time points after the start of IFN-β treatment. A full silencing effect of ISG15 translation was observed in cells exposed to siISG15 and treated daily with IFN-β, at 48 and 72 hours after induction of differentiation, with partial return of ISG15 protein to approximately 25–50% of baseline levels at later time points (Figure 2A). We therefore focused our studies on the 72 h and 96 h time points, reflecting maximal duration of ISG15 silencing (72 h) and the transition to return of partial ISG15 production (96 h).

ISG15 silencing had no effect on the appearance of IFN-β-treated cultures (Figure 2B) and left unchanged or even accentuated IFN-β-mediated reductions in myotube length, diameter, and area (Figure 2C). At 72 h, IFN-β combined with siISG15 treatment reduced myotube area 21%, not significantly different (p = 0.20) than the 10% reduction from IFN-β treatment alone. At 96 h, myotube area was reduced 45% with siISG15 treatment compared to 53% reduced from IFN-β alone (p = 0.74). Indeed, at the latest time point, 120 h, siISG15 and IFN-β treated cells were significantly more impaired (66% myotube area reduction) than IFN-β treatment alone (49% myotube area reduction, p = 0.02).These findings suggest that ISG15 does not mediate early C2C12 IFN-β myotoxicity.

## Discussion

Recent studies have identified type1 IFNs or their inducible gene products as biomarkers of DM [1]–[9], suggesting a mechanism of myofiber and capillary injury driven by type 1 IFNs. We therefore developed a cell culture model to explore the effects of type 1 IFNs on muscle cells.

We found that type 1 IFNs impair myotube differentiation of both C2C12 mouse myoblasts and human myoblasts. These findings are consistent with other studies demonstrating toxic and antiproliferative effects of type 1 IFNs in other cell lineages (endothelial cells and vascular smooth muscle) [17]–[21].

Regarding the mechanism of type 1 IFN myocyte toxicity, we focused on the potential role of ISG15, a gene whose transcript and protein are highly elevated in human DM muscle [1]–[6]. ISG15 is a member of the ubiquitin-like proteins family, with significant sequence homology to ubiquitin. Like ubiquitin, ISG15 is conjugated to many cellular proteins; the conjugation process requires the conjugating enzymes Ube1L, Ube2L6, and HERC5 as well as a deconjugating enzyme, USP18 [22]–[25]. Previous studies indeed demonstrated both the marked increase in free ISG15 protein as well as numerous ISG15 conjugated proteins in DM muscle and human skeletal muscle culture [5], suggesting a potential role as a mediator of IFN-mediated toxicity. We therefore examined the effect of IFN-α, IFN-β, and IFN-γ in the C2C12 model we developed and demonstrated that IFN-β, but not IFN-α, or IFN-γ, resulted in marked and sustained upregulation of ISG15. The lack of IFN-γ upregulation of ISG15 is explained by its known mechanism of action, as it binds to the gamma activation sequence (GAS) in promoter regions, and ISG15 does not contain this GAS element. In contrast, the known mechanisms of IFN-α and IFN-β involve binding to the interferon-stimulated regulatory element (ISRE), multiple copies of which are present in ISG15 [3]. However, as both IFN-α and IFN-β bind to a common receptor, the more potent and sustained upregulation of ISG15 by IFN-β is an empirical observation of unknown mechanism. Regardless of mechanism, we found that ISG15 silencing did not reverse IFN-β mediated toxicity, providing evidence against ISG15 being a major factor mediating the myotoxic effects of IFN-β.

Limitations of our studies include the use of an in vitro immortalized cell line model. Nevertheless, the molecular responses of this model to IFN-β are consistent with those found in numerous previously published studies of DM human tissue (blood, muscle, and skin) [1]–[12]. It is likely that other type 1 IFN induced genes are involved in myotoxicity, but the detailed mechanisms remain to be established. An approach similar to the one taken here, inhibiting other genes, might yield candidate mechanisms. However, currently no animal model of dermatomyositis exists, so that in vivo exploration of these mechanisms will likely await future development of such a model.

## Materials and Methods

### Cell Culture and Treatment with type 1 IFNs

Mouse skeletal muscle cells (C2C12; ATCC, Cat. No. CRL-1772) were cultured in 6-well plates, with growth medium consisting of DMEM (Atlanta Biologicals, Lawrenceville, GA) supplemented with 20% fetal bovine serum (Atlanta Biologicals, Lawrenceville, GA). Differentiation was initiated 72 h after seeding, by replacing the growth medium with D-MEM supplemented with 2% horse serum (Atlanta Biologicals, Lawrenceville, GA). At approximately 95% confluence, cells were treated daily with IFN-α or -β (PBL Interferon Source, Piscataway, NJ), at two different final concentrations (10 and 100 U/ml per well).

Human skeletal muscle cells (HuSK; ScienCell Research Laboratories, Cat no. 3500, Carlsbad, CA) were cultured in 6-well plates and treated with human IFN-β as previously described [2].

### siRNA Transfection

For small interfering RNA (siRNA) experiments, C2C12 myoblasts were transfected 24 h after seeding, when cell density was approximately 30–50%, with siRNA against ISG15 (Invitrogen, Carlsbad, CA) at the final concentration of 30 nM per well. Four to six hours after transfection, cells were treated daily with two different doses (10 and 100 U/ml) of mouse IFN-α or -β (PBL Interferon Source, Piscataway, NJ); differentiation was induced 48 hours later.

### Measurement of Length, Diameter, and Area of Newly Formed Myotubes

For quantitative morphological analysis, four micrographs were taken per well, at several time points, 48 h, 72 h, 96 h, and 120 h from the start of treatment) and for each, the length, diameter and area of the 10 visually largest myotubes were measured, using ImageJ 1.44 software (http://rsbweb.nih.gov/ij/, National Institutes of Health, Bethesda, MD). Statistical testing used nonparametric methods that do not assume Gaussian distributions of the data (Mann-Whitney tests).

### Western Blotting Analysis

Trypsinized cells were lysed in buffer containing 10 mM HEPES, 10 mM KCl, 1 mM EDTA, 0.1 nM EGTA, 10 mM DTT, 5 mM MgCl2 and Roche Complete Protease Inhibitor (Roche Diagnostic, Indianapolis, IN). Homogenates were sonicated and centrifuged for 10 min at 4°C (10,000 xg), supernatants were collected and protein contents were measured using BCA assay (Pierce Biotechnology, Rockford, IL). Samples were diluted in Loading Buffer 4X and denatured for 5 min at 95°C. Equal amounts of proteins were electrophoresed on 4–12% NuPage acrylamide gels (Invitrogen, Carlsbad, CA), and transferred onto nitrocellulose membranes. Membranes were blocked in Tris buffered saline with 0.1% Tween 20 (TBS-T) with 5% (wt/vol) nonfat milk powder and incubated overnight in the same solution containing rabbit polyclonal anti-ISG15 (Abcam, Cambridge, MA, cat # 45285; 1∶500 dilution), at 4°C, followed by a 1 hr incubation at RT with secondary antibody (HRP-conjugated goat anti-rabbit IgG Abcam, Cambridge, MA, Cat #6721, 1∶10,000 dilution). Immunoreactivity was detected by SuperSignal West Pico Maximum Sensitivity Substrate (Pierce Biotechnology, Rockford, IL) 5 min room temperature and exposed to film.
